# Machine Vision-Based Precision Detection of Circular Holes Using Canny Threshold Optimization and Zernike Moments

**DOI:** 10.3390/s26123699

**Published:** 2026-06-10

**Authors:** Juan Du, Jizheng Yu, Xintian Jiang, Xiaorui Li, Xiaodong Liu

**Affiliations:** School of Mechanical & Automotive Engineering, Liaocheng University, Liaocheng 252000, China; dujuan02@lcu.edu.cn (J.D.);

**Keywords:** circular hole detection, subpixel, Canny operator, Zernike moments, algorithm optimization

## Abstract

This study proposes a precision detection method that integrates Canny operator threshold optimization with Zernike moments to address the issue of low measurement accuracy associated with the manual inspection of circular holes in sheet metal during industrial testing. A complete automated measurement system was developed based on the MATLAB platform. First, adaptive median filtering is employed for image preprocessing, with superior performance in noise suppression and detail preservation validated through Peak Signal-to-Noise Ratio (PSNR) and Structural Similarity (SSIM) metrics. Subsequently, Otsu’s thresholding method achieves robust segmentation between target and background, laying the foundation for subsequent edge detection. An innovative adaptive threshold selection strategy for the Canny operator based on composite weight scoring was proposed during edge detection, significantly enhancing circular hole edges’ continuity and geometric integrity. Finally, by integrating Zernike moments with sub-pixel localization technology, ultra-precise localization of edge points at the sub-pixel level was achieved. Experimental results demonstrate that the system achieves a measurement repeatability standard deviation of less than 0.02 mm and controls the absolute error within ±0.05 mm.This performance surpasses the ±0.3 mm precision requirement in industrial settings, providing an effective solution for automated quality inspection of sheet metal hole manufacturing.

## 1. Introduction

Industrial manufacturing is undergoing a profound intelligent transformation, propelled by initiatives such as “Industry 4.0” and “Made in China 2025.” This evolution imposes increasingly stringent requirements for precision and efficiency in component processing [1]. Among various geometric features, circular holes’ dimensional and positional accuracy in sheet metal parts is particularly critical, as it directly impacts product performance, assembly quality, and structural integrity [2]. Traditional manual inspection methods, which rely on tools like calipers and optical projectors, are plagued by inherent limitations including low precision, subjective judgment, inefficiency, and poor data traceability, creating significant bottlenecks in modern production systems [3,4]. Consequently, the development of automated, high-precision, and efficient round hole inspection technology is urgently necessary for enhancing manufacturing intelligence and competitiveness [5].

Machine vision technology, characterized by its non-contact nature, high speed, and exceptional accuracy, presents an ideal solution to these challenges [6]. Integrating optical systems, image sensors, and advanced computational algorithms enables the rapid and objective acquisition of geometric information for precise measurement [7]. The detection of circular holes, a fundamental task in industrial vision, is a key indicator of the technological maturity in this domain [8]. While foundational theories in machine vision are often led by international research, domestic efforts have demonstrated remarkable progress in application-oriented innovations. Nevertheless, a significant challenge for both is bridging the gap between laboratory performance and reliability in real-world industrial settings [9,10].

Edge detection serves as the foundational step in most machine vision measurement pipelines. The technical trajectory has evolved from simple, general-purpose operators to sophisticated, specialized algorithms [11]. Early first-order differential operators, such as Sobel, Prewitt, and Roberts, remain relevant in applications where real-time performance is prioritized over high precision due to their computational simplicity [12]. However, their high sensitivity to noise, pixel-level localization accuracy, and tendency to produce fragmented edges render them unsuitable for high-precision metrology [13]. The Canny algorithm, introduced in 1986, represents a landmark achievement in edge detection theory [14]. Its comprehensive multi-stage process—Gaussian filtering, gradient calculation, non-maximum suppression, and dual-threshold hysteresis—was designed to optimally balance noise suppression with the preservation of edge details, solidifying its status as the industrial “gold standard” [15]. Despite its robustness, the algorithm’s performance in complex industrial environments is hindered by its parameter sensitivity. Selecting the Gaussian kernel’s variance (σ) and the dual thresholds requires a delicate and often subjective trade-off between denoising, detail retention, and edge continuity [16]. This limitation has spurred extensive research into more adaptive and robust solutions. Researchers worldwide have pursued diverse paths to enhance algorithmic adaptability. For instance, Boumaaz et al. [17] improved performance in noisy environments by integrating a dual-denoising mechanism into the Canny framework. In a groundbreaking deep learning approach, Li et al. [18] proposed the PiDiNet (Pixel Difference Network), which synergizes the differential principles of traditional operators with the hierarchical feature learning capabilities of deep convolutional networks, achieving superhuman edge detection accuracy while maintaining high inference speed.

Sub-pixel edge localization technology has been developed to surpass the resolution limit imposed by digital image sampling, enabling measurement accuracy at a finer than pixel level [19]. The primary technical routes include interpolation-based, fitting-based, and moment-based methods, each with distinct advantages for specific scenarios [20]. Interpolation-based methods, such as cubic spline or bilinear interpolation, estimate edge positions by constructing a continuous model of pixel intensity distribution. While computationally efficient, these methods generally offer limited accuracy [21]. Fitting-based approaches formulate a mathematical model of the edge profile and apply optimization algorithms like least squares to derive edge parameters. These can achieve high accuracy under ideal conditions but are often vulnerable to noise and outliers [22].

Among moment-based methods, Zernike moments are particularly notable for their robust mathematical foundation and excellent performance [23]. Defined as a complete set of orthogonal complex moments over the unit disk, they project image edge regions onto Zernike basis functions. By analyzing the amplitudes and phases across different orders, this method enables precise determination of sub-pixel edge position and orientation while [23] inherently providing rotation invariance and noise resilience [24]. Dong and Wang demonstrated a representative hybrid approach [25], which integrated an enhanced Sobel operator for initial pixel-level detection with a Zernike moments-based method for sub-pixel refinement, resulting in improved accuracy and fewer false edges compared to traditional algorithms. The pursuit of efficiency in sub-pixel positioning is also evident. Devin et al. [26] proposed a unified framework based on Zernike moments for sub-pixel positioning of edges and stripes in digital images. By introducing linear slopes and triangular wedge-shaped signal models, their method outperforms existing approaches in noisy environments. Cheng et al. [27] presented a sub-pixel edge detection algorithm based on Canny–Zernike moments. By combining the Canny detection algorithm and the Zernike moment method, the detection accuracy has been improved and the running time has been reduced. Li et al. [28] proposed a one-stage object detection framework named PDNet. By employing a prediction decoupling mechanism, the predictions for object categories and boundary positions were separated, thereby enhancing the detection performance.

At the application level, the technical approach for circular hole detection directly influences system performance and applicability. Methods based on the Hough transform and its improved variants (e.g., randomized and probabilistic Hough transforms) are notably robust to edge breakage and incomplete contours [29]. However, their computational complexity and memory consumption grow exponentially with the dimensionality of the parameter space, limiting their practicality for high-speed online detection [30]. The direct least squares circle fitting method adopts a different strategy, determining optimal circle parameters by minimizing the sum of squared distances from a set of edge points to the circle’s circumference [31]. This approach is computationally efficient but relies heavily on the precision and quality of the input edge points, making it sensitive to noise and outliers. Consequently, the prevailing paradigm in high-precision international measurement systems is a hybrid “sub-pixel edge localization + robust fitting” methodology [32,33]. This strategy leverages precise edge coordinates, obtained through techniques like Zernike moments, in conjunction with robust fitting algorithms to estimate geometric parameters, thereby striking an effective balance between accuracy and computational efficiency.

Despite significant advancements, translating machine vision-based round hole measurement from controlled laboratory settings to dynamic, complex industrial production lines remains challenging. To systematically address this challenge, this paper proposes an integrated and collaboratively optimized algorithm chain, designed to develop a high-precision, robust, and adaptive circular hole detection system for complex industrial environments. The core contributions of this work are twofold:

(1) Canny dual-threshold weighted optimization for geometric fidelity of circular holes Three dedicated evaluation metrics—circular hole closure, circularity, and contour closure—are established, and six weighted scoring functions (standard processing, hole priority, circularity priority, contour priority, balanced optimization, and adaptive balance) are designed. Through systematic traversal of 24 threshold configurations, the optimal dual thresholds (0.080,0.200) are automatically selected based on the weighted composite score, significantly improving edge continuity while maintaining a circularity of 0.9500.
(2) Adaptive subpixel edge localization based on multi-order Zernike moment synergy A multi-order collaborative strategy is adopted, led by the (n=3,m=1) order and supplemented by the (n=1,m=1) and (n=2,m=0) orders. Combined with a coarse-to-fine two-stage radius scanning mechanism that minimizes the $Z_{31}$ moment magnitude, five radius estimation methods are integrated, and an adjustment factor is introduced to compensate for systematic bias, thereby significantly enhancing subpixel localization accuracy.

The remainder of this paper is structured as follows. Section 2 achieves feature region extraction by selecting optimal image denoising and segmentation algorithms. Section 3 establishes the Canny optimization algorithm framework. Section 4 performs precise fitting of circular holes using adaptive Zernike moments and least squares methods. Section 5 presents experimental results. Section 6 concludes the paper.

## 2. Image Preprocessing and Segmentation

### 2.1. Image Preprocessing

During image acquisition and transmission, interference such as salt-and-pepper noise and Gaussian noise is often unavoidably introduced. This leads to degraded image quality and consequently affects the accuracy of subsequent analysis and measurement. Therefore, noise suppression in image preprocessing is critical in ensuring detection precision.

Standard mean filtering methods exhibit some noise reduction capabilities, but they tend to blur edges during processing and offer limited suppression of salt-and-pepper noise. Gaussian filtering achieves smoothing through weighted averaging, yet its fixed convolution kernel parameters restrict its adaptive suppression of impulse noise. In contrast, as a nonlinear filtering method, adaptive median filtering dynamically adjusts the filter window size based on local image characteristics. This approach effectively removes impulse noise while better preserving image edges and detailed information. Its mathematical expression is shown in Equation (1):

(1) $$g\left(x,y\right)=\mathrm{Median}\left\{f\left(i,j\right)|\left(i,j\right)\in S_{xy}\right\}$$

In the formula, g(x,y) is the filtered pixel, *Median* denotes the operation of taking the middle value after sorting, f(i,j) is the original pixel, and $S_{xy}$ is the neighborhood window.

**Figure 1:**
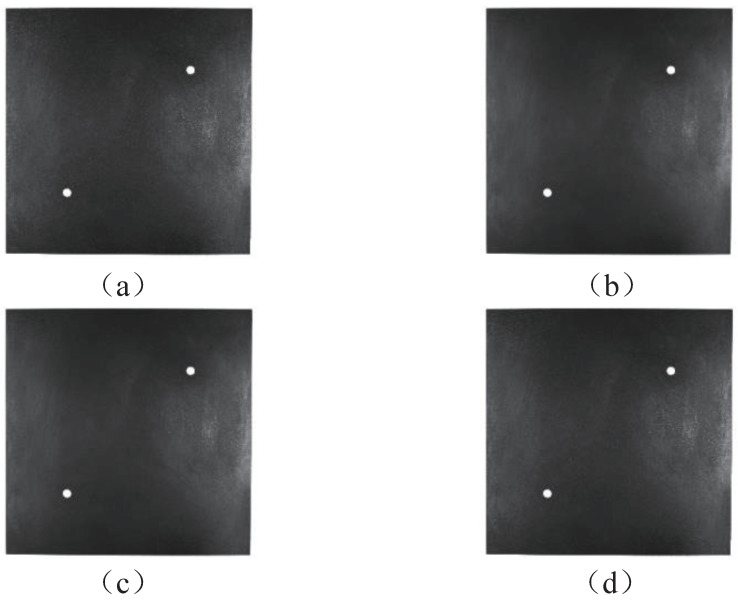
Comparison of Filtered Images. (**a**) Original Grayscale Image; (**b**) Mean Filter (5 × 5); (**c**) Gaussian Filter (σ=1.5); (**d**) Adaptive Median Filter (maximum window size 5 × 5).

To validate the practical effectiveness of various filtering methods, experiments were conducted on mean filtering (5 × 5 window), Gaussian filtering (σ=1.5), and adaptive median filtering (maximum window 5 × 5). The results are shown in Figure 1. Visually, adaptive median filtering better preserves edge structures and detail integrity while reducing noise.

Peak Signal-to-Noise Ratio (PSNR) and Structural Similarity (SSIM) are introduced as assessment metrics to quantify the evaluation of filtering performance further. The results are shown in Table 1. PSNR reflects the signal-to-noise ratio between the denoised and original images, with higher values indicating lower distortion. SSIM measures the image’s ability to preserve structure, brightness, and contrast; values closer to 1 indicate better structural retention.

As shown in Table 1, the adaptive median filter achieved a PSNR of 40.72 dB and an SSIM of 0.958, with both metrics significantly outperforming the other two methods. This demonstrates its distinct advantages in both noise suppression and structural preservation. The Gaussian filter exhibited intermediate performance, balancing smoothing and edge preservation. In contrast, the mean filter resulted in severe detail loss due to excessive smoothing, yielding the lowest values for both PSNR and SSIM.

Based on the combined visual and quantitative evaluation results, adaptive median filtering demonstrated the best noise reduction and detail preservation performance. Therefore, this method was selected as the filtering algorithm for the image preprocessing stage.

### 2.2. Threshold Segmentation

To accurately separate the workpiece from the background in the filtered grayscale image, this paper employs threshold segmentation to binarize the image, enabling effective extraction of the target workpiece [34]. Given the characteristics of the workpiece images in this study—uniform illumination and grayscale distribution close to binary—three typical threshold segmentation methods were compared: the iterative threshold method achieves stable segmentation through dynamic threshold adjustment; the Otsu method automatically selects the global optimal threshold based on the principle of maximizing interclass variance, offering excellent standardization and automation properties; while the optimal parameter adaptive thresholding method, though locally adaptive, is sensitive to noise and prone to oversegmentation.

**Figure 2:**
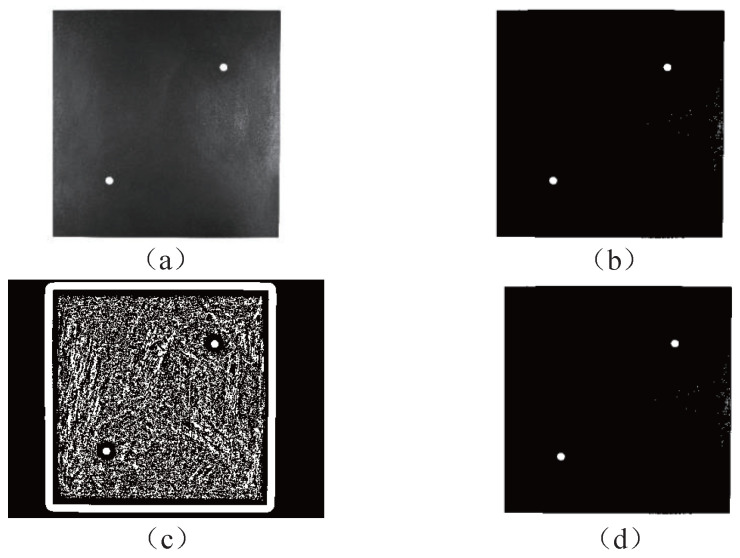
Experimental results of threshold segmentation. (**a**) the adaptive median-filtered image; (**b**) Iterative Thresholding (T=140.7982); (**c**) Optimized Adaptive Thresholding (Window 111, C=0.00); (**d**) Otsu’s method (T=140.0001).

The experimental results of the three methods are shown in Figure 2, with their performance parameters compared in Table 2. The results indicate that the iterative thresholding method and the Otsu method exhibit consistent performance on key metrics such as the number of target pixels, the number of connected regions, and the average compactness, both significantly outperforming the adaptive thresholding method. Given that the Otsu method offers stronger standardization and automation advantages while maintaining high segmentation quality, this approach is ultimately selected as the implementation solution for threshold segmentation in this paper.

Experimental results indicate that the iterative thresholding method and Otsu’s thresholding method exhibit consistent performance on key parameters and outperform adaptive methods. Considering Otsu’s thresholding method offers higher levels of standardization and automation, this approach was ultimately selected for threshold segmentation processing in this study.

### 2.3. Feature Region Localization

Building upon image segmentation, this study employs connected component analysis, region attribute filtering, and region of interest (ROI) extraction techniques to localize target circular holes and workpiece regions precisely. The specific workflow is as follows: First, the filtered and enhanced image undergoes binarization using the Otsu method; Next, a morphological opening operation using a circular structural element is performed to eliminate fine noise, followed by a closing operation to connect fragmented edges and ensure regional integrity. Subsequently, noise regions are filtered out based on area attributes, retaining target regions with distinct features. Finally, combining connected component analysis with ROI extraction completes the localization and extraction of the workpiece’s feature region. The definition of the feature region is shown in Equation (2):

(2) $$D=\max\{B_{1},B_{2},B_{3},\ldots ,B_{n}\}$$

In the formula, *D* represents the number of pixels in the largest connected region, i.e., the one with the most significant area among all connected regions, denoting the “working feature region”; $B_{i}$ denotes the number of pixels in the *i*-th connected region (i.e., the area of that connected region), where i=1,2,…,n; *n* represents the total number of connected regions (i.e., the quantity of all connected domains marked in the image).

**Figure 3:**
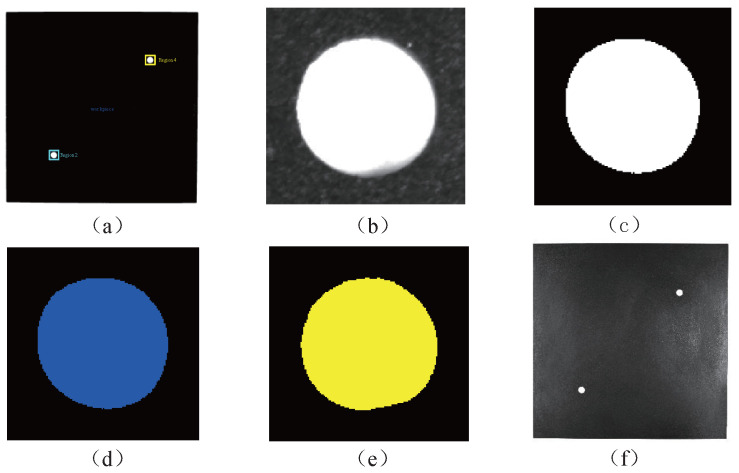
Results of Feature Region Calibration Experiment. (**a**) Overall Workpiece Feature Region Localization; (**b**) Original Circular Hole Image ROI; (**c**) Binarized circular hole image ROI; (**d**) ROI Marking of Feature Region 2; (**e**) ROI Marking of Feature Region 4; (**f**) Original Grayscale Image.

After adaptive median filtering and Otsu threshold segmentation, the experimental results for feature region localization are shown in Figure 3. The figure clearly demonstrates the localization effect of the entire workpiece area and the ROI extraction results for each circular hole region. Experimental measurements indicate compactness values of 1.0109 and 1.0066 for circular hole regions 2 and 4, respectively, confirming the extracted areas’ regular shapes and high integrity. The precise calibration of feature regions achieves effective separation between target and background and establishes a reliable foundation for subsequent accurate geometric parameter measurements.

## 3. Edge Detection and Optimization

After completing image preprocessing, it is necessary to extract edge features from the image further. The edge information obtained is crucial in measuring circular hole dimensions and fitting outer contours.

### 3.1. Canny Edge Detection Algorithm

Edge detection serves as the foundation of dimensional measurement. Among common operators, the Roberts operator is simple and fast yet sensitive to noise, whereas the Sobel and Prewitt operators provide better noise suppression but yield wider edges and weaker responses to oblique edges. In contrast, the Canny algorithm achieves superior noise suppression and edge continuity via its multi-stage processing [35].

**Figure 4:**
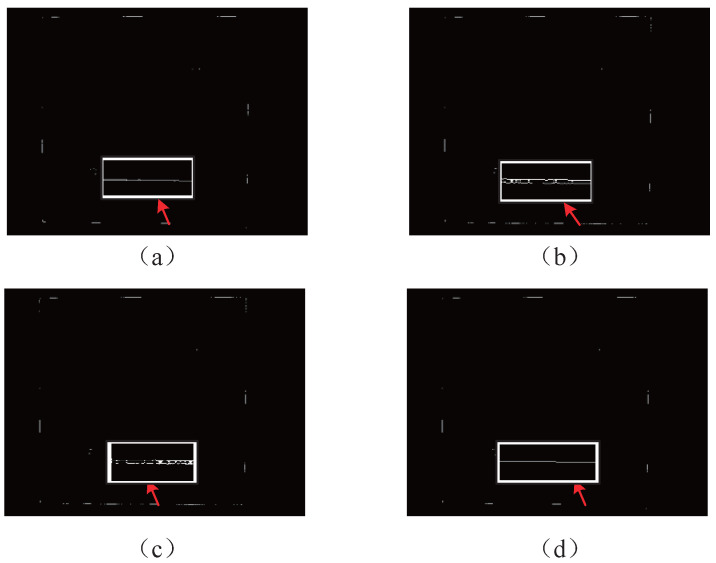
Experimental results of various edge detection algorithms. (**a**) Edges Detected by the Roberts Operator (12,406 Pixels); (**b**) Edges Detected by the Sobel Operator (15,429 Pixels); (**c**) Edges Detected by the Prewitt Operator (15,691 Pixels); (**d**) Edges Detected by the Canny Operator (11,968 Pixels).

The experimental results of the four edge detection algorithms are shown in Figure 4. The Roberts algorithm detected 12,406 pixels, with a single-line outer contour but poor straightness. The Sobel algorithm detected 15,429 pixels, featuring a double-line outer contour with irrelevant vertical line connections but good straightness. The Prewitt algorithm detected 15,691 pixels, with thicker irrelevant vertical lines between the double lines compared to Sobel, yet maintaining good straightness; the Canny algorithm detected 11,968 pixels, featuring a single-line outer contour with the best circularity of the hole edges, demonstrating superior overall performance compared to the other three algorithms.

**Figure 5:**
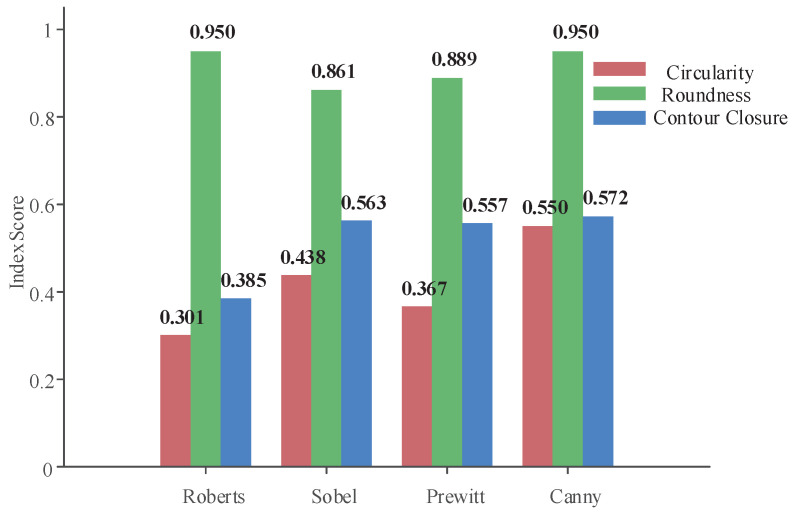
Comparison of Performance Parameters for Various Edge Detection Algorithms.

Due to the high pixel density of the images, the edges must be magnified in the MATLAB R2021b display window to clearly observe the details. To objectively evaluate the performance of various edge detection algorithms, this paper introduces three quantitative evaluation metrics: circular hole closure (the ratio of the number of edge pixels actually detected to the theoretical closed perimeter, reflecting the continuity and integrity of the edge), circularity of the circular hole (the deviation from the ideal radius, measured as the standard deviation of the distances from the edge points to the center of the fitted circle, reflecting the degree of conformity between the edge shape and an ideal circle), and contour closure (the ratio of the convex hull perimeter of the outer contour’s connected component to the actual edge perimeter, reflecting the completeness of the outer contour). The algorithms were quantitatively compared based on these three metrics, and the results are shown in Figure 5.

Analysis indicates that the Canny algorithm delivers optimal circularity metrics (0.9500) performance, demonstrating the highest alignment between its edge contours and ideal circles. Simultaneously, it significantly outperforms the Roberts algorithm (0.301) in evaluating hole closure (0.550). Therefore, edges extracted by the Canny algorithm are more suitable for sub-pixel level detection requirements, providing a more accurate pixel foundation for subsequent circular hole fitting and diameter measurement. Furthermore, the dual-threshold mechanism employed by the Canny algorithm offers a more flexible detection range and demonstrates clear advantages in edge recognition accuracy. Consequently, this paper selects the Canny algorithm as the edge detection method.

Implementing the Canny edge detection algorithm primarily involves the following key steps. First, the algorithm applies Gaussian filtering to smooth the image, suppress high-frequency noise in the original image, and reduce the generation of false edges. Subsequently, building upon the denoising process, the algorithm employs the Sobel operator to compute the gradient components in horizontal and vertical directions [36]. This yields each pixel’s gradient magnitude and direction, providing the foundation for subsequent edge localization and direction analysis.The relevant formulas are shown in Equations (3)–(6).

(3) $$P_{x}\left(x,y\right)=\left(f\left(x,y+1\right)-f\left(x,y\right)+f\left(x+1,y+1\right)-f\left(x+1,y\right)\right)/2$$

(4) $$P_{y}\left(x,y\right)=\left(f\left(x,y+1\right)-f\left(x+1,y+1\right)+f\left(x,y\right)-f\left(x+1,y\right)\right)/2$$

(5) $$M\left(x,y\right)=\sqrt{P_{x}{\left(x,y\right)}^{2}+P_{y}{\left(x,y\right)}^{2}}$$

(6) $$\theta \left(x,y\right)=\arctan\left(P_{y}\left(x,y\right)/P_{x}\left(x,y\right)\right)$$

In the equation, $P_{x}\left(x,y\right)$ and $P_{y}\left(x,y\right)$ are the horizontal and vertical gradients at (x,y). M(x,y) is the edge strength, θ(x,y) is the gradient direction, f(x,y) is the grayscale value, and (x,y) are the pixel coordinates.

The Canny algorithm incorporates a non-maximum suppression (NMS) operation to achieve finer edges. This step compares neighboring pixels along gradient directions, retaining only local maxima points. It significantly refines edge widths, eliminates redundant edge bands, and produces more accurate and distinct detection results.

Finally, the Canny algorithm employs a dual-threshold detection and connectivity strategy to classify pixels into three categories: firm edges, weak edges, and non-edges. Strong edge pixels are directly output as final edges, while weak edge pixels are retained only if connected to firm edges; otherwise, they are suppressed. This approach effectively eliminates false edges caused by noise, significantly enhancing the accuracy and continuity of edge detection.

### 3.2. Threshold Optimization

This paper proposes a threshold optimization method based on weighted comprehensive evaluation to address the sensitivity issues associated with dual-threshold settings in the Canny operator. The method systematically assesses the impact of various threshold configurations on edge detection quality by defining six sets of weight combinations with distinct preferences. The weight combinations are specifically configured as follows:

[0.33,0.33,0.33]: Standard processing, balanced weighting across all metrics;

[0.7,0.15,0.15]: Round hole priority, emphasizes round hole closure;

[0.15,0.7,0.15]: Roundness priority, emphasizes roundness metrics;

[0.15,0.15,0.7]: Contour priority, emphasizing contour closure;

[0.4,0.3,0.3]: Balanced optimization, slightly favoring circular hole closure;

Adaptive balance: Employing a dynamic weight adjustment strategy.

The three values within each weighting group correspond sequentially to the evaluation weights for circular hole closure, roundness, and contour closure. Based on this, four threshold groups with a total of 24 configuration schemes have been established:

Low-threshold group: [0.01,0.05]; [0.02,0.08]; [0.03,0.12]; [0.05,0.15]; [0.08,0.2]; [0.1,0.25] (emphasizes capturing weak edges);

Medium Threshold Group: [0.01,0.1]; [0.02,0.15]; [0.03,0.2]; [0.05,0.25]; [0.08,0.3]; [0.1,0.35] (balances edge continuity);

High Threshold Group: [0.05,0.2]; [0.1,0.25]; [0.15,0.3]; [0.2,0.4]; [0.25,0.5]; [0.3,0.6] (filtering non-primary edges);

Balanced Threshold Group: [0.02,0.1]; [0.05,0.15]; [0.08,0.2]; [0.1,0.25]; [0.15,0.3]; [0.2,0.3] (comprehensive balanced characteristics).

**Figure 6:**
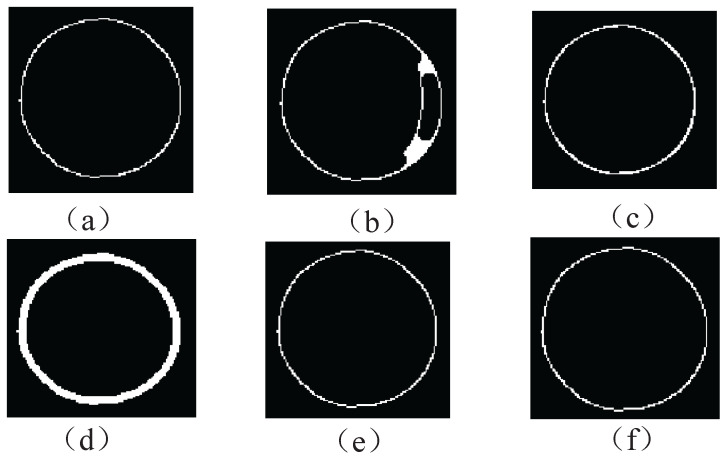
Magnified view of the circular hole using the Canny edge detection optimized parameter algorithm. (**a**) Standard Processing; (**b**) Hole Priority; (**c**) Circularity Priority; (**d**) Contour Priority; (**e**) Balancing Optimization; (**f**) Adaptive Balance.

Figure 6 displays the enlarged edges of circular holes after various weighting rebalancing treatments, visually illustrating the impact of different threshold strategies on edge quality.

**Figure 7:**
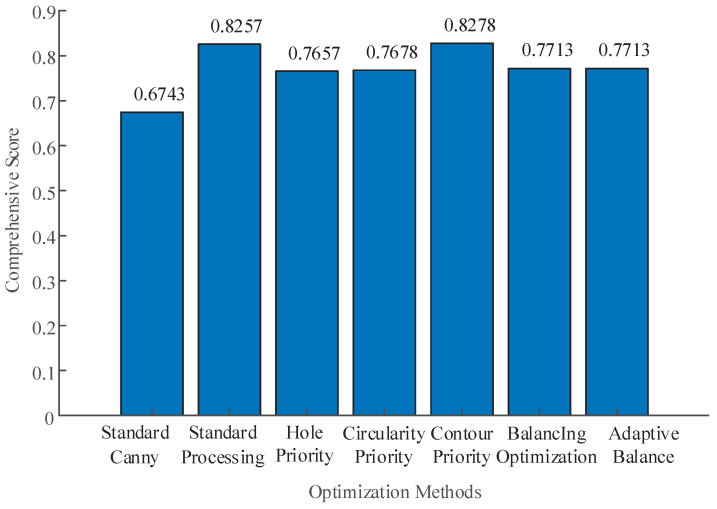
Canny threshold weighted calculation for composite score.

Figure 7 shows the comprehensive scores for each configuration, calculated through weighted averaging of three metrics—hole closure, circularity, and contour closure. The specific scores are as follows: Standard Canny scored 0.6743, Standard Processing scored 0.8257, Circle Hole Priority scored 0.7657, Roundness Priority scored 0.7678, and Contour Priority scored 0.8278, while Balanced Optimization and Adaptive Balance both scored 0.7713.

**Figure 8:**
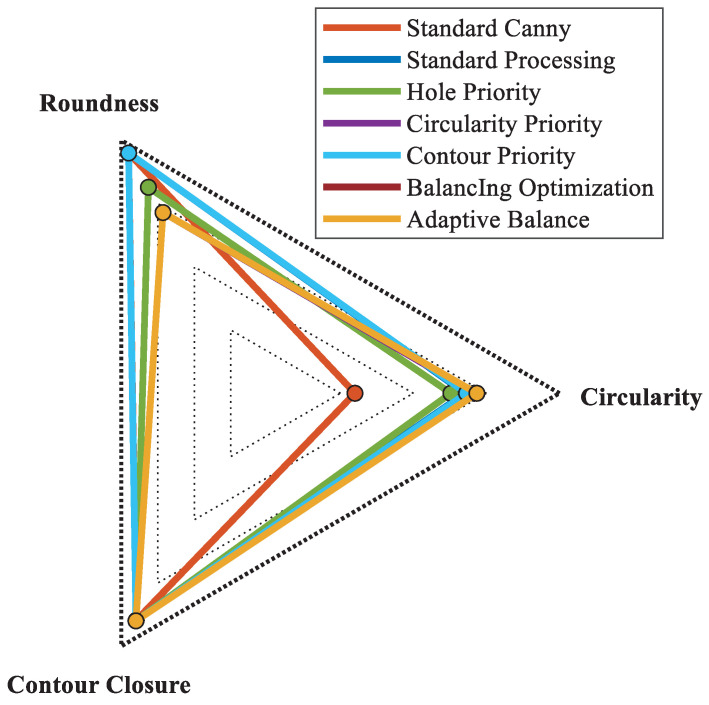
Optimized Weighted Processing for Six Sets of Individual Metric Charts.

The specific performance of each weighting scheme across the three evaluation metrics is shown in Figure 8. Analysis reveals that while the contour-priority group achieved the highest overall score (0.8278), its larger edge width is disadvantageous for subsequent Zernike-based sub-pixel edge detection. In contrast, the standard processing group matched the contour-priority group in roundness (0.9500) while exhibiting finer edge widths, making it more suitable for high-precision measurement requirements. Therefore, the Canny dual-threshold parameters (0.080, 0.200) corresponding to standard processing were ultimately selected as the optimal configuration.

## 4. Subpixel Localization and Circular Fitting

### 4.1. Adaptive Optimization Algorithm for Subpixel Edge Detection Based on Zernike Moments

Adaptive optimization algorithms based on Zernike moments enable sub-pixel-level localization of image edges, significantly enhancing detection accuracy. Compared to traditional algorithms that can only localize to the pixel level, sub-pixel technology achieves finer estimation through the moment method, least-squares fitting, and interpolation. Among these, the moment method, though computationally complex, is widely adopted due to its robust theoretical foundation and excellent rotational invariance.

(1) Conventional Zernike Moment Subpixel Algorithm

Zernike moments are a class of orthogonal complex moments defined within the unit circle, exhibiting strong invariance to image rotation [37]. The *n*th-order *m*th-degree Zernike moment is defined as shown in Equation (7), with its corresponding Zernike basis function given by Equation (8):

(7) $$Z_{nm}=\frac{n+1}{\pi }\underset{x^{2}+y^{2}\leq 1}{\;\int \int \;}f\left(x,y\right)V_{nm}^{*}\left(\rho ,\theta \right)\;dx\;dy$$

(8) $$V_{nm}\left(\rho ,\theta \right)=R_{nm}e^{jm\theta }$$

In the equation, $Z_{nm}$ is the Zernike moment of order *n* and degree *m*.$V_{nm}^{*}\left(\rho ,\theta \right)$ is the complex conjugate of the basis function. *n* is the moment order. *m* is the repetition count, satisfying |m|≤n and n−|m| being even. f(x,y) is the grayscale value at (x,y); $\rho =\sqrt{x^{2}+y^{2}}$ is the polar radius, θ is the polar angle, where $x^{2}+y^{2}\leq 1$ confines the integral to the unit circle.

(2) Adaptive Optimization Algorithm Based on Zernike Moment Subpixel Edge Detection

**Figure 9:**
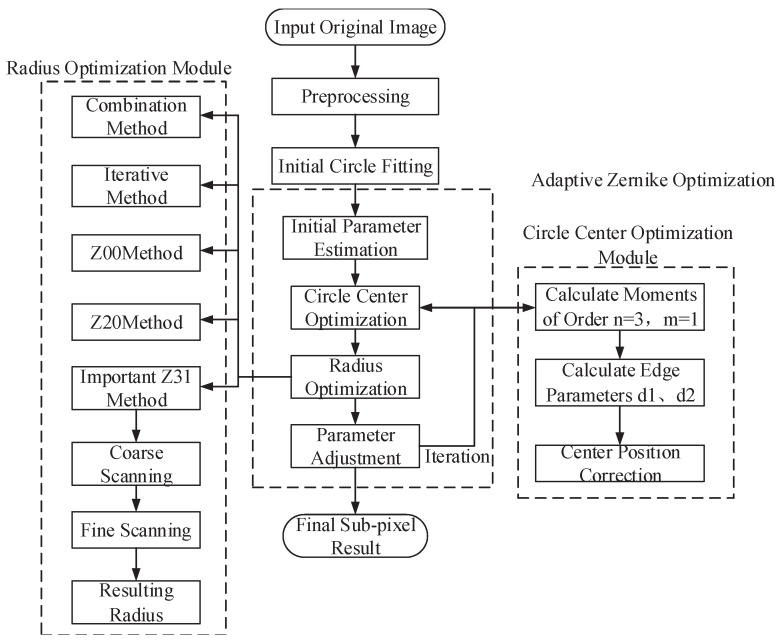
Overall Flowchart of the Zernike Moment Adaptive Algorithm.

This study proposes a novel adaptive Zernike moment edge-fitting method to detect complex edges precisely. Its core comprises two major modules: adaptive circle fitting and multi-strategy radius optimization. The algorithm flow is illustrated in Figure 9.

This study employs an edge parameter-based precise calculation model for adaptive Zernike circle fitting. By recursively computing Zernike moments using Equations (9)–(14), it sequentially solves for offset, amplitude, phase values, and sub-pixel displacement. Subsequently, it corrects the circle center coordinates, achieving high-precision estimation of parameters $d_{1}$ and $d_{2}$.

(9) $$d_{1}=\frac{2\cdot |Z_{31}{|}^{2}}{|Z_{11}|}\cdot \cos\left(\phi_{31}-3\phi_{11}\right)$$

(10) $$d_{2}=\frac{2\cdot |Z_{31}{|}^{2}}{|Z_{11}|}\cdot \sin\left(\phi_{31}-3\phi_{11}\right)$$

(11) $$|Z_{mn}|=\sqrt{{\left(Z_{mn}^{re}\right)}^{2}+{\left(Z_{mn}^{im}\right)}^{2}}$$

(12) $$\phi_{nm}=\arctan\left(\frac{Z_{nm}^{im}}{Z_{nm}^{re}}\right)$$

(13) $$\Delta x=d_{1}\cdot \cos\left(\theta \right)-d_{2}\cdot \sin\left(\theta \right)$$

(14) $$\Delta y=d_{1}\cdot \sin\left(\theta \right)+d_{2}\cdot \cos\left(\theta \right)$$

In the equation, $d_{1}$ and $d_{2}$ are edge parameters representing the characteristic position parameters of the circular edge region, measured in pixels; $Z_{31}$ and $Z_{11}$ denote the third-order first-degree and first-order first-degree Zernike moments, with average errors of 0.068 pixels and 0.109 pixels, respectively; $Z_{nm}$ denotes the amplitude value of the Zernike moment; $\phi_{nm}$ represents the phase in radians; $Z_{nm}^{re}$ and $Z_{nm}^{im}$ denote the real and imaginary parts of the Zernike moment, respectively; Δx and Δy denote the sub-pixel-level offsets of the edge point in the *x* and *y* directions, respectively, in pixels; θ denotes the polar angle of the edge point in radians.

For circular radius estimation, the study further developed a collaborative optimization framework integrating five complementary strategies: an optimization algorithm based on $Z_{31}$ moment magnitude, the $Z_{20}$ method based on circularity metrics, the normalized $Z_{00}$ method, an iterative convergence method, and a weighted combination method utilizing multiple moment information. The $Z_{31}$ method theoretically minimizes the $Z_{31}$ moment magnitude during optimal circular fitting, determining the optimal radius through multi-radius scanning:

(15) $$r_{\mathrm{opt}}=\arg\min_{r\in [r_{\min},r_{\max}]}\left|Z_{31}\left(r\right)\right|$$

In the equation, $r_{\mathrm{opt}}$ denotes the optimized optimal radius; argmin represents “the variable that minimizes the expression,” i.e., finding the *r* value that minimizes the subsequent expression; $r\in [r_{\min},r_{\max}]$ defines the search range, indicating that *r* lies between the minimum and maximum radii; $|Z_{31}\left(r\right)|$ denotes the absolute value of the amplitude of the Zernike moment of order n=3 and m=1 at a given radius *r*.

Experimental results demonstrate that this framework (particularly the n=3, m=1-order method) achieves outstanding performance in sub-pixel center localization, with an average error of only 0.068 pixels, significantly outperforming conventional approaches.

To further enhance the estimation accuracy of the radius parameter, this paper employs a progressive search strategy combining multi-radius scanning with two-stage coarse-to-fine optimization. This strategy adopts a global-local collaborative search mechanism:

First, a coarse multi-radius scan is performed to screen the optimal interval, as shown in Equation (16):

(16) $$R_{\mathrm{coarse}}=r_{i}|r_{i}=r_{\mathrm{init}}\cdot \alpha_{i},\alpha_{i}\in \left[0.9,1.1\right]$$

In the formula, $R_{\mathrm{coarse}}$ represents the set of radius candidates in the coarse scanning phase; $r_{i}$ denotes the candidate radius value; $r_{\mathrm{init}}$ represents the initial radius estimate; $\alpha_{i}$ is the scaling factor, ranging from 0.9 to 1.

Finally, perform a detailed scan as shown in Equation (17):

(17) $$R_{\mathrm{fine}}=\left\{r_{i}|r_{i}\in \left[r_{\mathrm{best}}-\Delta r,r_{\mathrm{best}}+\Delta r\right]\right\}$$

In the formula, $R_{\mathrm{fine}}$ represents the set of radius candidates in the fine scanning stage; $r_{i}$ denotes the candidate radius value; $r_{\mathrm{best}}$ indicates the optimal radius found in the coarse scanning stage; and Δr is the step size for fine scanning.

**Figure 10:**
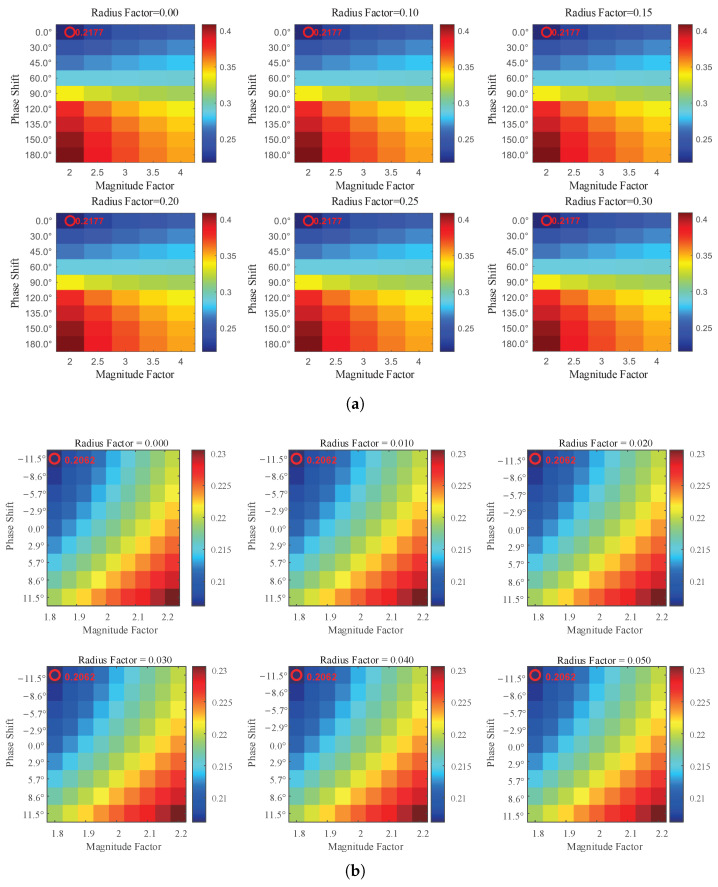
Adaptive Zernike parameter optimization. (**a**) Heatmap matrix showing errors under different parameters; (**b**) Detailed search of error heatmap matrices under different parameters.

The final selection uses the radius value that minimizes the $Z_{31}$ amplitude as the optimized result. Figure 10 illustrates the parameter optimization process.

In this error heatmap matrix, colors represent the degree of parameter adaptation: blue regions indicate good adaptation, while green regions indicate poor adaptation. Based on this, the study first conducted a coarse search within the range of [2,4] for the amplitude factor, preliminarily determining the radius factor to be 0.2177. Subsequently, the amplitude factor range was narrowed to [1.8,2.2] for a fine search, ultimately optimizing the radius factor further to 0.2062.

Regarding order selection, a multi-moment synergistic strategy was established, primarily based on n=3 and m=1, supplemented by n=1 and m=1 (error 0.109 pixels) and n=2 and m=0 (error 0.131 pixels). To further integrate the complementary advantages of different moments, a weighted combination approach was adopted to aggregate the contributions of multiple moments, as expressed in Equation (18):

(18) $$r_{\mathrm{combined}}=r_{Z31}\cdot \left(1+\delta_{\mathrm{adjust}}\right)$$

In the formula, $r_{\mathrm{combined}}$ represents the final optimized radius calculated by the combined method; $r_{Z_{31}}$ represents the base optimized radius calculated using the $Z_{31}$ method; $\delta_{\mathrm{adjust}}$ is the adjustment factor used to fine-tune the results of the $Z_{31}$ method.

Experimental validation demonstrates that the conventional method achieves a mean error of 0.454 pixels on the standardized test set. In contrast, the optimized adaptive Zernike method reduces the error to 0.096 pixels under favorable experimental conditions, representing a 4.7-fold improvement in accuracy with a significant advantage. This combined strategy effectively enhances the algorithm’s adaptability and robustness across diverse edge features.

**Figure 11:**
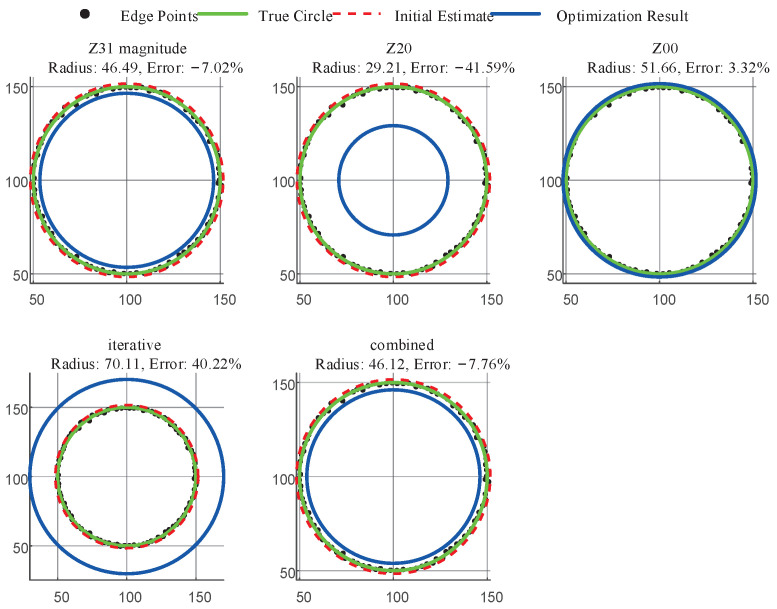
Visualization of Zernike Radius Optimization.

**Figure 12:**
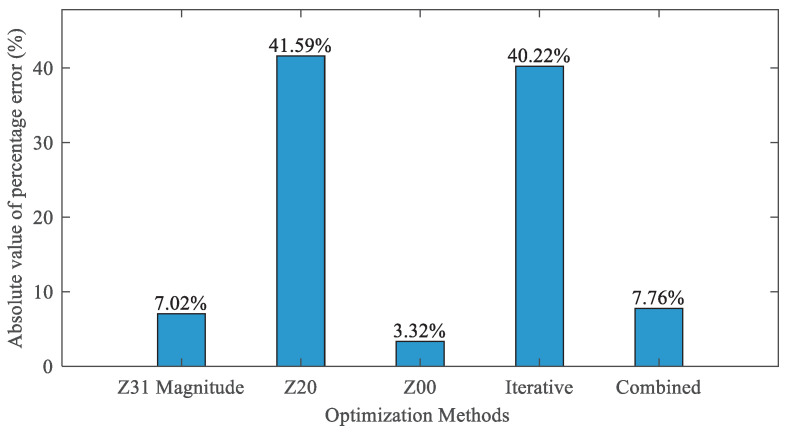
Error Analysis of Radius Optimization Method.

The performance advantage of this method in sub-pixel edge detection is further validated through Zernike radius optimization visualization results, as shown in Figure 11 and Figure 12.

The measurement results for the same standard 50 mm circular aperture using each method are as follows: Z31 calculated radius of 46.49 mm, relative error of −7.02%; Z20 yielded a radius of 29.21 mm with a relative error of −41.59%; Z00 produced a radius of 51.66 mm with a relative error of 3.32%; the iterative method resulted in a radius of 70.11 mm with a relative error of 40.22%; and the combined method produced a radius of 46.12 mm with a relative error of −7.76%. Multiple repeated experiments demonstrate that, influenced by noise randomness, the measurement errors of each method exhibit normal fluctuations within a reasonable range. This characteristic effectively simulates the uncertainty factors present in real industrial measurement environments, further validating the robustness and accuracy of the proposed method in practical applications.

Based on the above analysis, the radius optimization module designed in this paper significantly enhances the calculation accuracy of circular hole radii by systematically comparing the performance of various algorithms, including the combination method, iterative method, $Z_{00}$, $Z_{20}$, and $Z_{31}$. This provides reliable technical assurance for workpiece diameter measurement.

### 4.2. Least Squares Fitting of Circles

Precisely extracting circular hole edges is a critical step in workpiece quality inspection. Its core lies in accurately identifying and extracting discrete point sets from the workpiece edges, which serve as the data foundation for circle fitting. This paper employs the least squares method to fit the edges of circular holes in sheet metal workpieces [38]. This approach determines optimal fitting parameters by minimizing the sum of squared errors, thereby minimizing the overall deviation between the fitted circle and the actual edge points. The mathematical expression for the standard circle is shown in Equation (19):

(19) $$R^{2}={\left(x-A\right)}^{2}+{\left(y-B\right)}^{2}$$

In the Equation, *R* denotes the radius of the circle, i.e., the distance from the center to any point on the circle; (x,y) represents the coordinates of any point on the circle; *A* denotes the horizontal coordinate of the center; *B* denotes the vertical coordinate of the center.

For numerical convenience, the standard form is expanded and reparameterized as follows:

(20) $$\left\{\begin{array}{c} a=-2A\\ b=-2B\\ c=A^{2}+B^{2}-R^{2} \end{array}\right.$$

Equation (20) can be simplified as shown in Equation (21):

(21) $$x^{2}+y^{2}+ax+by+c=0$$

The core of achieving a precise fit for circular holes lies in solving for parameters *a*, *b*, and *c*, thereby deriving the center coordinates and radius. To accomplish this, based on the set of edge points $\left\{\left(x_{i},y_{i}\right)|i\in \left(1,2,3,\ldots ,N\right)\right\}$, the following fitting model is established:

First, define the Euclidean distance from each edge point to the circle’s center, expressed as:

(22) $$d_{i}^{2}={\left(x_{i}-A\right)}^{2}+{\left(y_{i}-B\right)}^{2}$$

In the formula, $d_{i}$ denotes the Euclidean distance from the *i*-th edge point to the center of the circle; $\left(x_{i},y_{i}\right)$ represents the coordinates of the *i*-th edge point.

Based on this, the squared Euclidean distance residual from each observation point to the target circle is defined as:

(23) $$\begin{array}{cc} \delta_{i}=d_{i}^{2}-R^{2} & ={\left(x_{i}-A\right)}^{2}+{\left(y_{i}-B\right)}^{2}-R^{2}\\ \; & =x_{i}^{2}+y_{i}^{2}+ax_{i}+by_{i}+c \end{array}$$

In the equation, $\delta_{i}$ denotes the squared deviation of the *i*-th edge point from the fitted circle; $d_{i}^{2}$ represents the squared distance of the *i*-th edge point from the circle center; $R^{2}$ indicates the squared radius of the fitted circle.

To evaluate the overall fitting quality, the objective function Q(a,b,c) is constructed as the sum of squared deviations for all edge points, as shown in Equation (24):

(24) $$Q\left(a,b,c\right)=\Sigma \delta_{i}^{2}=\sum {\left(x_{i}^{2}+y_{i}^{2}+ax_{i}+by_{i}+c\right)}^{2}$$

In the equation, $\Sigma \delta_{i}^{2}$ represents the sum of squared deviations from all edge points to the fitted circle; $\Sigma {\left(x_{i}^{2}+y_{i}^{2}+ax_{i}+by_{i}+c\right)}^{2}$ denotes the sum of squared deviations for all points.

To find the parameter combination that minimizes the objective function, take the partial derivatives for the three parameters *a*, *b*, and *c* and set them equal to zero, as shown in Equation (25):

(25) $$\begin{array}{cc} \frac{\partial Q(a,b,c)}{\partial a} & =\sum 2\left(x_{i}^{2}+y_{i}^{2}+ax_{i}+by_{i}+c\right)x_{i}=0\\ \frac{\partial Q(a,b,c)}{\partial b} & =\sum 2\left(x_{i}^{2}+y_{i}^{2}+ax_{i}+by_{i}+c\right)y_{i}=0\\ \frac{\partial Q(a,b,c)}{\partial c} & =\sum 2\left(x_{i}^{2}+y_{i}^{2}+ax_{i}+by_{i}+c\right)=0 \end{array}$$

In the equation, $\frac{\partial Q(a,b,c)}{\partial a}$ denotes the partial derivative of the objective function *Q* with respect to parameter *a*; $\frac{\partial Q(a,b,c)}{\partial b}$ denotes the partial derivative of the objective function *Q* with respect to parameter *b*; $\frac{\partial Q(a,b,c)}{\partial c}$ denotes the partial derivative of the objective function *Q* with respect to parameter *c*.

After mathematical transformations and simplifications, intermediate variables are introduced as shown in Equation (26):

(26) $$\begin{array}{cc} C & =\left(N\sum x_{i}^{2}-\sum x_{i}\sum x_{i}\right)\\ D & =\left(N\sum x_{i}y_{i}-\sum x_{i}\sum y_{i}\right)\\ E & =\left(N\sum x_{i}^{3}+N\sum x_{i}y_{i}^{2}-\sum \left(x_{i}^{2}+y_{i}^{2}\right)\sum x_{i}\right)\\ G & =\left(N\sum y_{i}^{2}-\sum y_{i}\sum y_{i}\right)\\ H & =\left(N\sum x_{i}^{2}y_{i}+N\sum y_{i}^{3}-\sum \left(x_{i}^{2}+y_{i}^{2}\right)\sum y_{i}\right) \end{array}$$

In the equation, *N* represents the total number of sample points, i.e., the quantity of edge points participating in the fitting; $C=(N\sum x_{i}^{2}-\sum x_{i}\sum x_{i})$ denotes a matrix element related to all *x*-coordinates, reflecting the dispersion of *x*-coordinates; $D=(N\sum x_{i}y_{i}-\sum x_{i}\sum y_{i})$ represents the correlation term between *x* and *y* coordinates, embodying the covariance of the point set in the xy direction; $E=(N\sum x_{i}^{3}+N\sum x_{i}y_{i}^{2}-\sum \left(x_{i}^{2}+y_{i}^{2}\right)\sum x_{i})$ is a higher-order mixed term used for subsequent parameter calculations, reflecting the distribution characteristics of the point set along the *x*-axis; $G=(N\sum y_{i}^{2}-\sum y_{i}\sum y_{i})$ represents the matrix element related to all *y*-coordinates, reflecting the dispersion of the *y*-coordinates; $H=(N\sum x_{i}^{2}y_{i}+N\sum y_{i}^{3}-\sum \left(x_{i}^{2}+y_{i}^{2}\right)\sum y_{i})$ is a higher-order mixed term used for subsequent parameter calculations, reflecting the distribution characteristics of the point set in the *y*-direction.

From this, the parameter expression can be derived as:

(27) $$\begin{array}{cc} a & =\frac{HD-EG}{CG-D^{2}}\\ b & =\frac{ED-HC}{CG-D^{2}}\\ c & =-\frac{\sum \left(x_{i}^{2}+y_{i}^{2}\right)+A\sum x_{i}+B\sum y_{i}}{N} \end{array}$$

The geometric parameter expression for the circular hole is ultimately derived as shown in Equation (28):

(28) $$A=-\frac{a}{2},\;B=-\frac{b}{2},\;R=1/\left(2\sqrt{a^{2}+b^{2}-4c}\right)$$

**Figure 13:**
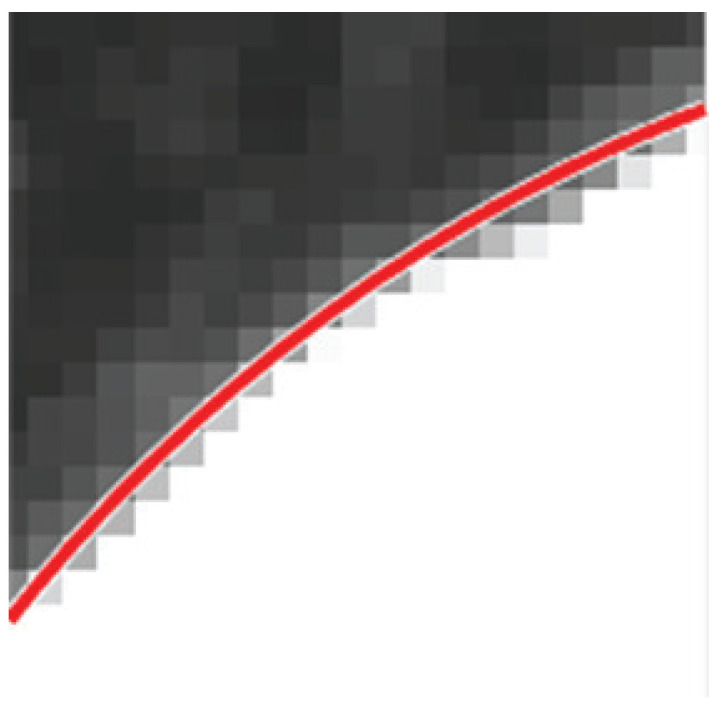
Subpixel Fitting of Circular Hole.

**Figure 14:**
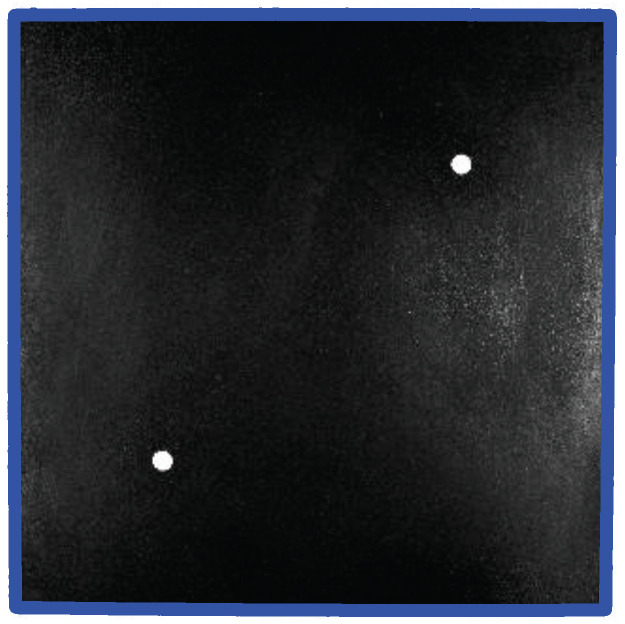
Panel Outer Contour Fitting Diagram.

The subpixel fitting results for circular holes in sheet metal using least squares combined with conventional moments are shown in Figure 13:

As shown in Figure 13, the red curve represents the extracted edge fitting result. Its continuity and alignment with the grayscale gradient trend reflect the algorithm’s boundary recognition accuracy at the junction between continuous grayscale transition zones and high-brightness regions. Building upon the sub-pixel-level edge fitting of the circular hole, precise fitting of the workpiece’s outer contour is required to obtain its complete shape information. As the representation of the workpiece’s overall form, the fitting accuracy of the outer contour significantly impacts the final measurement and analysis results. This paper employs the Hough transform method for outer contour recognition, which effectively extracts the primary boundary features of the workpiece and is particularly suitable for detecting contours of regular geometric shapes such as rectangles and squares. This paper further integrates connected component analysis to address scenarios where the outer contour signal is weak or partially missing in specific images. Selecting the region with the most significant area and most regular shape as the main contour of the workpiece effectively suppresses stray noise interference and enhances the robustness of contour extraction. The outer contour fitting results are shown in Figure 14, demonstrating that this method can accurately restore the geometric shape of the workpiece, providing a reliable basis for subsequent dimensional measurement and shape analysis.

## 5. Experiment and Analysis

### 5.1. Camera Calibration

To meet the high-precision measurement requirements for sheet metal hole making, camera calibration and lens distortion correction are performed using an improved Zhang’s calibration method.

First, chessboard images are acquired from multiple orientations. Sub-pixel corner detection is applied to extract interior corner points, and statistical filtering is employed to remove outliers. Then, based on the pinhole imaging model, the homography matrix for each image is solved, followed by a closed-form solution of the camera intrinsic parameters (focal length and principal point) and extrinsic parameters (rotation matrix and translation vector).

Subsequently, three radial distortion coefficients $k_{1}$, $k_{2}$, $k_{3}$ and two tangential distortion coefficients $p_{1}$, $p_{2}$ are introduced to construct the distortion model. The radial correction formula is given as:

(29) $$\begin{array}{c} \delta_{xr}=x\left(k_{1}r^{2}+k_{2}r^{4}+k_{3}r^{6}+K\right)\\ \delta_{yr}=y\left(k_{1}r^{2}+k_{2}r^{4}+k_{3}r^{6}+K\right) \end{array}$$

The tangential correction formula is given as:

(30) $$\begin{array}{cc} \delta_{xd} & =2p_{1}xy+p_{2}\left(r^{2}+2y^{2}\right)+K\\ \delta_{yd} & =2p_{1}\left(r^{2}+2y^{2}\right)+2p_{2}xy+K \end{array}$$

In the equation, (x,y) are the normalized coordinates in the original image, and $\left(\delta_{xd},\delta_{yd}\right)$, $\left(\delta_{xr},\delta_{yr}\right)$ are the actual distortion coordinates. Finally, an iterative optimization procedure is employed to minimize the reprojection error, resulting in an average reprojection error controlled within 0.25 pixels. All intrinsic and extrinsic parameters, along with the distortion coefficients, are then output.

After completing the calibration, an inverse distortion mapping is applied to each measured image to generate a distortion-free corrected image. Subsequently, the pixel coordinates are sequentially transformed into the camera coordinate system and then into the world coordinate system, thereby obtaining the physical dimensions of the circular holes.

Potential error sources include the flatness error of the calibration board, sensor noise and quantization error of the camera, as well as insufficient richness in the number or poses of calibration images, which may lead to unstable parameter estimation. To ensure measurement traceability and accuracy reliability, the system employs a metrologically certified standard chessboard calibration board. All calibration parameters are recorded in a configuration file, which can be quickly loaded before each measurement, and periodic recalibration is performed to monitor parameter drift.

### 5.2. Experimental Platform

**Figure 15:**
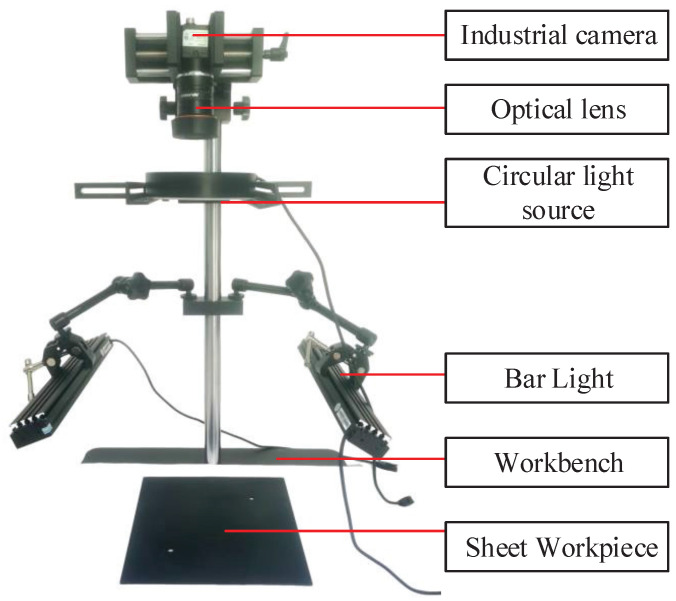
Experimental Physical Environment Diagram.

This study established a comprehensive machine vision inspection platform to validate the feasibility and accuracy of the sheet material hole quality inspection system. This platform integrates a high-resolution industrial camera, specialized optical lenses, adjustable ring and bar light sources, a precision workbench, and standard sheet material workpieces, creating a stable and controllable image acquisition environment. Test panels measuring 200 mm × 200 mm were selected for experimentation. These panels featured uniformly distributed circular holes with a diameter of 7 mm, whose inner diameter machining precision was strictly controlled within ±0.3 mm. This provided a reliable reference standard for system error assessment.The platform’s hardware configuration is illustrated in Figure 15.

Regarding software systems, this study leverages the MATLAB platform for system development, capitalizing on its strengths in rapid algorithm prototyping. Combined with its extensive image processing and computer vision toolboxes, it achieves fully automated processing from image acquisition and preprocessing to feature extraction and dimensional measurement.

### 5.3. Results and Analysis

**Figure 16:**
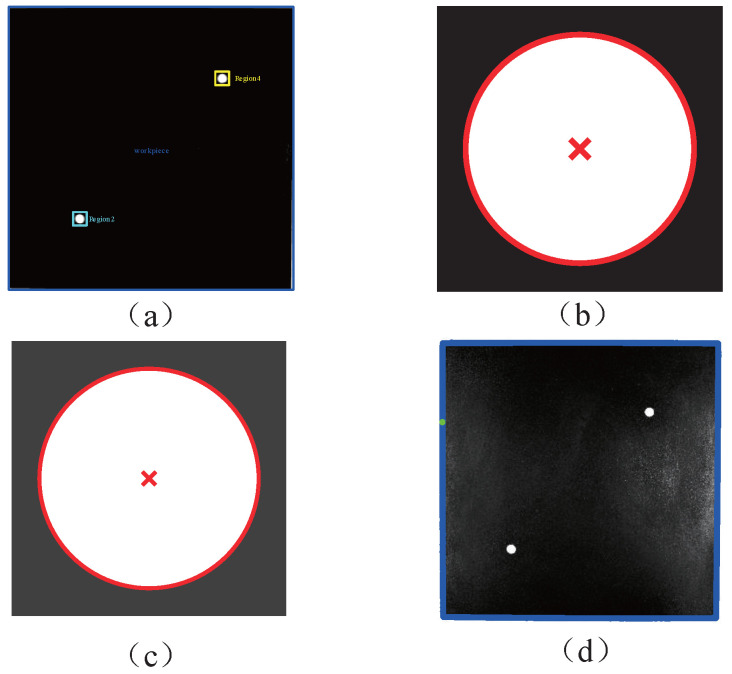
Analysis Results. (**a**) Overall Workpiece Feature Region Localization; (**b**) Results of Canny Edge Detection and Circular Hole Fitting for Region 2; (**c**) Results of Canny Edge Detection and Circular Hole Fitting for Region 4; (**d**) Workpiece Outer Contour Extraction Results.

This section systematically evaluates the proposed detection method’s comprehensive performance across three dimensions: visualization quality, measurement accuracy, and repeatability. The system supports autonomous configuration of key analytical parameters and outputs complete visual results, including feature region localization, Canny edge detection with circular hole fitting, and workpiece outer contour extraction, as shown in Figure 16.

Visualization results demonstrate that the proposed method performs satisfactorily across all processing stages. Figure 16a illustrates the precise localization of feature regions on the workpiece, clearly delineating multiple regions of interest (ROIs) to provide a reliable foundation for subsequent local hole detection. In Figure 16b,c, edges extracted by the Canny algorithm exhibit continuous integrity, with circular fitting curves closely matching actual edges and accurate center positioning. Figure 16d demonstrates clear and continuous extraction of the outer contour, effectively suppressing noise interference and establishing a solid foundation for overall dimensional analysis of the workpiece.

**Figure 17:**
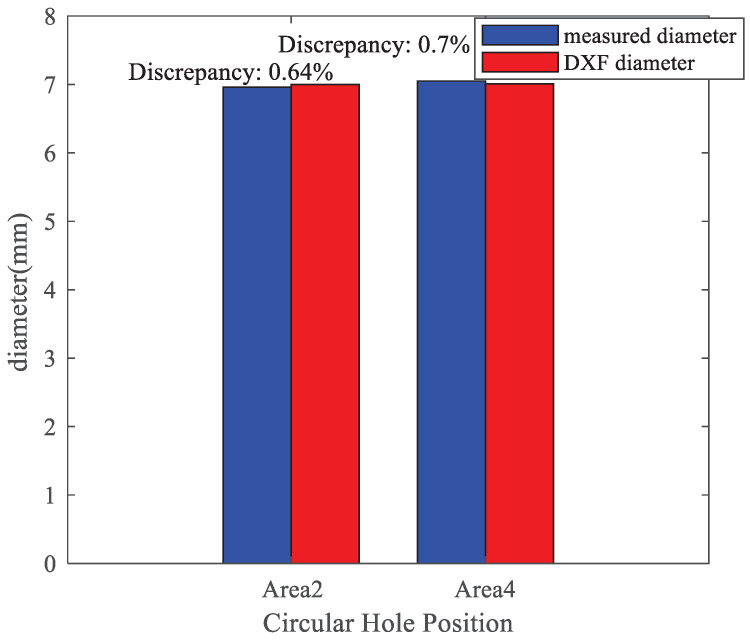
Comparison of Round Hole Measurement Results with DXF.

Building upon the qualitative assessment, random circular holes within regions 2 and 4 of the test plate were selected for diameter measurement to quantify further the system’s measurement consistency and robustness across the entire plate. The results are presented in Table 3 and Figure 17.

Data analysis indicates that the measured diameters in both regions deviate only minimally from the theoretical design value (7 mm). The diameter in Region 2 is 6.9550 mm, with an absolute error of 0.0450 mm and a relative error of 0.64%. The diameter in Region 4 is 7.0490 mm, with an absolute error of 0.0490 mm and a relative error of 0.70%. The relative error for both sets of measurements was below 1%, verifying the system’s ability to maintain stable measurement accuracy across different locations.

Eight repeated measurements were conducted on the standard circular hole (Φ7.000 mm) in Region 2 to thoroughly evaluate system stability, with results shown in Table 4. Data indicates an absolute error range of 0.0145–0.0459 mm, where the maximum absolute error represents only 15.3% of the preset industrial accuracy (±0.3 mm). The relative error ranged from 0.2071% to 0.6557%, with an average relative error of 0.417%. Notably, the error standard deviation reached 0.0126 mm, a metric demonstrating the system’s excellent repeatability and measurement stability.

To verify the measurement consistency and accuracy of the proposed inspection system for different production batches and different hole diameters, plates with nominal diameters of Φ7 mm and Φ10 mm were selected for the experiment. For each diameter, five test plates were randomly sampled from three independent production batches (Batch A, B, and C). Each plate contained four circular holes, resulting in a sample size of 3×5×4=60 holes per diameter, and a total of 120 holes for both diameters. All workpieces were fabricated using the same material and machining process to eliminate the influence of material variability on the measurement results.

For each combination of hole diameter and batch, the average measured diameter, mean absolute error, maximum absolute error, and sample standard deviation were calculated based on the 20 holes (5 plates × 4 holes). The results are presented in Table 5.

The data show that for both Φ7 mm and Φ10 mm hole diameters, the mean absolute error for each batch is less than 0.042 mm, and the sample standard deviation for all groups does not exceed 0.014 mm, both of which are far superior to the industrial standard requirement of ±0.3 mm. For the same nominal diameter, the maximum difference in the mean measured diameter among different batches is only 0.0059 mm, indicating that the system exhibits excellent batch-to-batch stability. These results demonstrate that the proposed method maintains high measurement consistency across different hole diameters and production batches.

The comprehensive experimental results demonstrate that this detection system performs well in assessing the quality of holes drilled in sheet materials. In qualitative analysis, the system achieves precise calibration of feature regions, continuous extraction of sub-pixel edges, and high-precision circular hole fitting, validating the effectiveness and reliability of the algorithm design. In quantitative evaluation, the absolute error in circular hole diameter measurement did not exceed 0.05 mm, with relative errors consistently below 1%, demonstrating outstanding single-measurement precision. More importantly, the minimal error fluctuation range and low standard deviation observed in repeatability experiments confirm the system’s repeatability and stability, meeting industrial field requirements.

## 6. Conclusions

This paper addresses the challenges of low measurement accuracy and insufficient efficiency in manual inspection of circular holes in sheet metal during industrial testing by developing a precision inspection system based on machine vision. Through systematic algorithm optimization and innovation, adaptive median filtering is employed during image preprocessing to suppress noise interference while preserving detailed features effectively. Otsu’s thresholding method is applied during image segmentation to separate workpieces and backgrounds precisely. An adaptive threshold optimization method based on the Canny operator with composite weight scoring is proposed in the edge detection stage, significantly enhancing edge continuity and geometric fidelity. Finally, combining Zernike moment sub-pixel localization technology enables the precise positioning of edge points at an ultra-pixel level. Experimental validation demonstrates that the system achieves circular hole diameter measurement accuracy better than ±0.05 mm, with a repeatability standard deviation below 0.0126 mm. All performance metrics significantly exceed the ±0.3 mm precision requirement for industrial applications. This research provides a comprehensive technical solution for automated inspection of sheet metal hole quality, exhibiting notable advantages in enhancing measurement accuracy and holds significant engineering application value.

## Figures and Tables

**Table 1:** PSNR and SSIM Values.

Filtering Method	PSNR (dB)	SSIM
Mean Filter	34.4841	0.844
Gaussian Filter	35.799	0.88
Adaptive Median Filter	40.7249	0.958

**Table 2:** Comparison of Performance Parameters for Threshold Segmentation Methods.

Segmentation Method	Number of Target Pixels	Number of Connected Regions	Average Compactness
Iterative Thresholding	4,637,334	3	0.7612
Optimal Adaptive Thresholding	3,952,252	2417	0.6676
Otsu Thresholding	4,637,334	3	0.7612

**Table 3:** Round Hole Measurement Results Compared with DXF.

Position	Measured Diameter (mm)	DXF Diameter (mm)	Absolute Error (mm)	Relative Error (%)
Region: 2	6.9550	7	0.0450	0.6429
Region: 4	7.0490	7	0.0490	0.7000

**Table 4:** Repeat Measurement Data for Region 2.

Measurement No.	Measured Diameter (mm)	Absolute Error (mm)	Relative Error (%)
1	6.9753	0.0247	0.3529
2	6.9840	0.0160	0.2286
3	6.9550	0.0450	0.6429
4	7.0380	0.0380	0.5429
5	6.9541	0.0459	0.6557
6	7.0145	0.0145	0.2071
7	6.9740	0.0260	0.3714
8	7.0213	0.0213	0.3043

**Table 5:** Measurement error statistics for different hole diameters and production batches (data rounded to four decimal places).

Nominal Diameter (mm)	Production Batch	Number of Holes (*n*)	Mean Measured Diameter (mm)	Mean Absolute Error (mm)	Standard Deviation (mm)
Φ7	Batch A	20	6.9678	0.0322	0.0112
Φ7	Batch B	20	6.9709	0.0291	0.0103
Φ7	Batch C	20	6.9650	0.0350	0.0119
Φ7 (all batches)		60	6.9679	0.0321	0.0113
Φ10	Batch A	20	9.9617	0.0383	0.0131
Φ10	Batch B	20	9.9586	0.0414	0.0140
Φ10	Batch C	20	9.9642	0.0358	0.0125
Φ10 (all batches)		60	9.9615	0.0385	0.0132

## Data Availability

The data presented in this study are available upon request from the corresponding author.
